# Core Values of Family Physicians and General Practitioners in the African Context

**DOI:** 10.3389/fmed.2021.667144

**Published:** 2021-05-31

**Authors:** Henry J. O. Lawson, David N. N. Nortey

**Affiliations:** ^1^Department of Community Health, University of Ghana, Accra, Ghana; ^2^Korle Bu Teaching Hospital, Accra, Ghana

**Keywords:** core value, Africa, family physician, comprehensive care, collaborative care, continuity of care, patient centered care, lifelong learning

## Abstract

**Introduction:** Family Medicine is a growing specialty in the medical world. While it is expected that the specialty should have its own unique and established core values and tasks, the breath of the practice in several countries of the world has made this a daunting task. Core values and Tasks have far reaching effects on professions. They guide development of curricula, methods of instruction, standards of performance and even the culture of the profession. We aimed to explore the core value system of Family Physicians and General Practitioners practicing in Africa.

**Methods:** Using the Delphi technique, a purposive selection of African Family Medicine practitioners in academia, public service, private practice and clinical training across Central, East, North, South and West Africa was conducted. Participants were asked to select five core values from an alphabetically collated global list of 29 core values in an online survey. The five most selected core values were collated and sent out in the second round to the participants to rank in order of importance.

**Results:** Practitioners from nine African countries in three out of the five United Nations subregions of Africa completed the study. The first round of the study saw participation of a team of nineteen experts who selected the following five core values—Comprehensive care, Continuity of Care, Collaborative Care, Patient centered care, and Life-long learning.

**Discussion/Conclusion:** The core values selected were not very different from global literature. These core values should guide the development of curricula, standardization of training methods and creation of benchmarks for standards of practice for the specialty in Africa.

## Introduction

Family Medicine is a specialty which is well-established in Europe, Australia, Canada, and North America; and is gradually expanding in South America, Asia, and Africa. Like all other medical specialties, it is expected that Family Medicine should have its own unique core values and tasks. Unfortunately, the wide breath of the practice of Family Medicine globally and the low number of countries where it is a recognized specialty has made this a daunting task.

Core values are the fundamental beliefs of an individual, organization or a group of people with a common goal or like-mindedness. They consist of a set of statements, phrases, or typically carefully selected words that undergird the actions, behaviors and mindset of the entities involved; as well as dictate the modalities by which things are done, and cut to the core of ideologies fundamental to the individuals involved. Typical core values include loyalty, integrity, and honesty.

Different regions of the world need to define their core values and tasks to dovetail into a global definition of core values and tasks for Family Physicians and General Practitioners. Core values come in handy not only during the curative work, but also during roles in preventive care, health promotion, community mobilization, research, leadership, and resource allocation.

Research from different parts of the world have documented region-specific core values and tasks. For example, patient-centeredness, holism and empathy have been listed in the United Kingdom (1), while practice management, wellness medicine, information technology, home visits, family dynamics, and community medicine are the five core values for family physicians in the United States (2).

Family Medicine is a relatively new discipline in sub-Saharan Africa. Few countries have established training programmes and even fewer are recognized by governments and incorporated in the healthcare system (3). The situation is more grave in francophone and lusaphone countries. Is imperative that a clear definition of the core values and tasks be developed to support integration of the specialty into African countries. A study conducted in Sudan reported leadership, team work, continuity of care, comprehensive care, and adherence to national clinical guidelines as core values for Family Medicine in that country (4).

There is a paucity of data on core values and tasks for family physicians in the African context. We therefore aimed to engage African experts and practitioners in Family Medicine and General Practice to assist in identifying five (5) core values and tasks for Family Medicine in the African Context.

## Methods

This study used qualitative design and the Delphi technique (5). We searched the scientific literature to identify publications on Family Medicine core values from different regions worldwide. Core values and tasks were identified from Sudan, Netherlands, Scandinavia, United Kingdom, United Arab Emirates, and the United States of America (1, 2, 4, 6–8).

**Figure 1:**
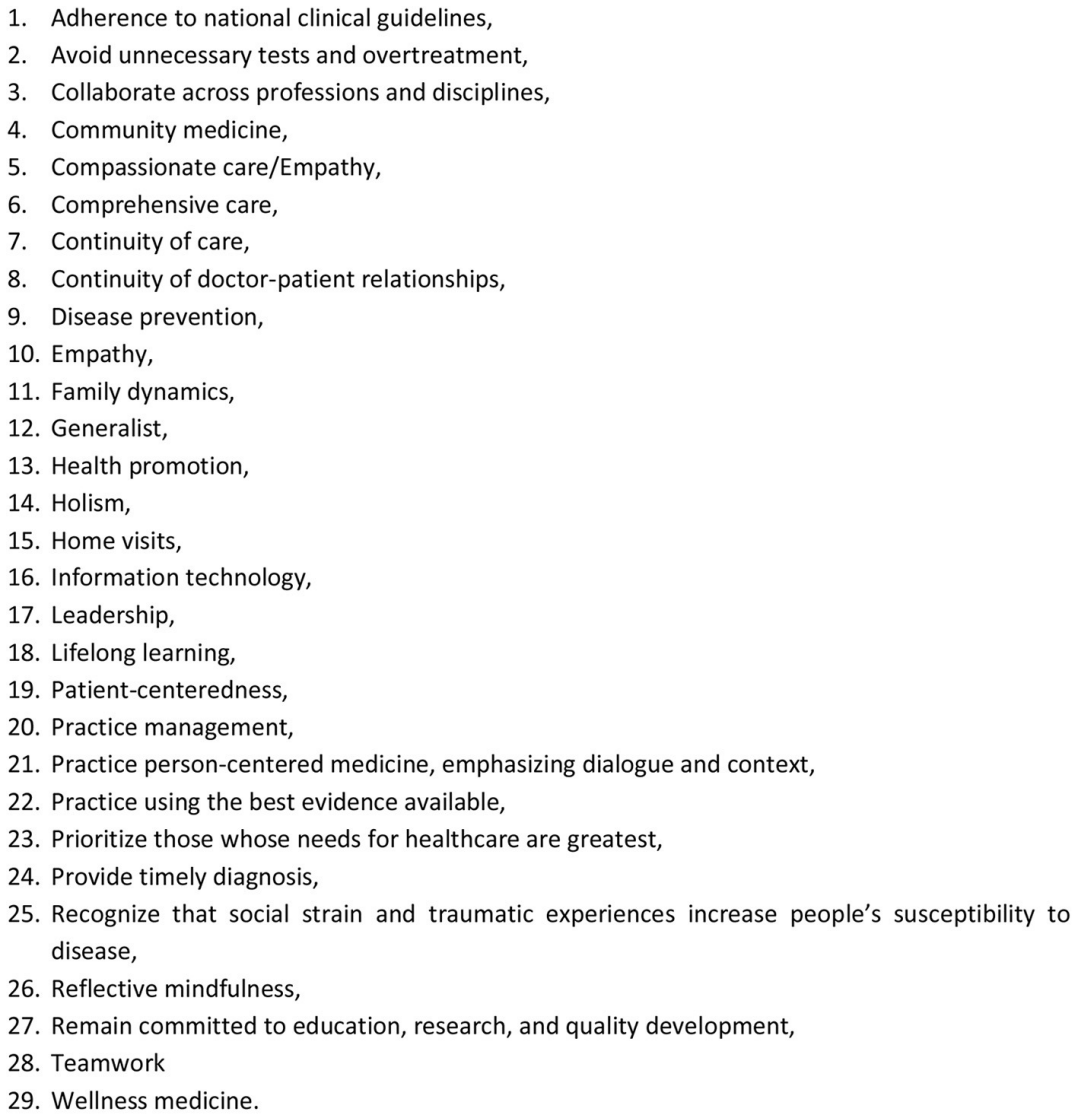
Checklist of Collated Core Values.

**Figure 2:**
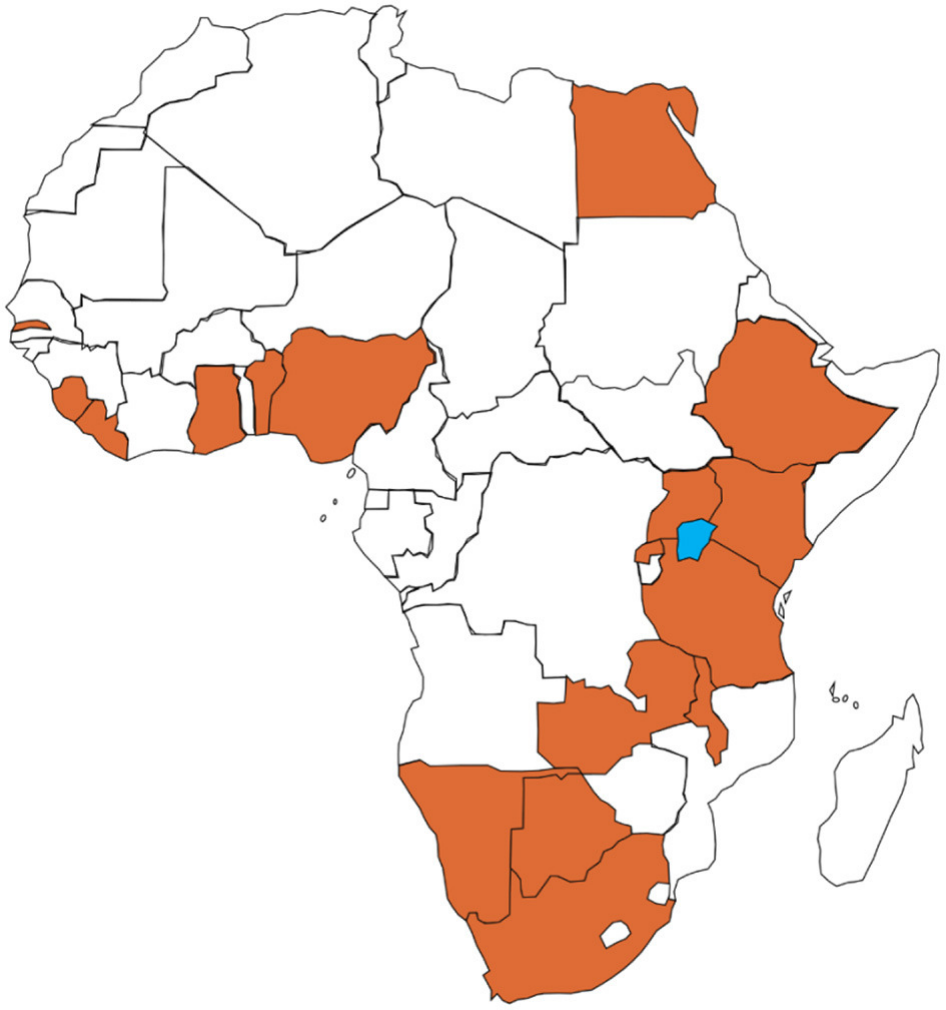
Map of Africa showing Countries with participating Family Physicians.

Through this search, we developed a list of 29 core values relevant to the practice of Family Medicine (Figure 1). This list was developed into an online survey using Google forms. In the first round of the survey, 29 purposefully selected Family Physicians or General practitioners working in academia, public service, private practice and clinical tutors from 16 countries across four of the five United Nations sub-regions of Africa (9) were invited to participate in an online survey (Figure 2). A listserv from the Africa Region of the World Organization of Family Doctors (WONCA) was deployed to reach out to countries within the sub-region who had any activity in Family Medicine.

The core values were listed in alphabetical order and were not ranked in any other way. Each respondent was asked to select five values that they believed to be most critical for Family Medicine in the African context. Participants were given the freedom to merge any of the core values listed and make comments. The result of the selections was collated, and the core values were ranked according to the highest number of times each particular core value was selected. The core values which had the highest number of selections by respondents were recorded. In the second round of the study, the five most selected options from the first round were listed alphabetically and sent out to respondents. They were asked to rank these five selected choices in order of importance to the practice of a Family Physician or General Practitioner in the African context.

## Results

Twenty-nine physicians were approached in round one, 20 responded however one of the responders declined to participate. The non-response rate was 34.5%. Nineteen participants thus completed this round from the following countries—Ethiopia (1), Ghana (5), Liberia (1), Nigeria (5), Rwanda (1), Sierra Leone (1), South Africa (2), The Gambia (1), and Uganda (2). The Male: Female ratio was 1.7:1.0; with ages from 35 to 73years. Eleven out of 19 respondents (57.9%) had completed their terminal qualification in Family Medicine (See Table 1).

**Table 1:** Demographic characteristics of respondents.

	**Round 1**	**Round 2**
**Age (in Years)**		
35–40	4	1
41–45	3	3
46–50	5	–
51–55	2	1
56–60	–	–
61–65	2	–
66–70	2	–
>70	1	–
**Gender**		
Male	12	2
Female	7	3
**Years of Practice after Housemanship**		
1–10	4	1
11–20	9	3
>20	6	1
**Years of Practice as a Family Physician**		
1–10	12	4
11–20	5	1
>20	2	0
**Practice Setting**		
Urban	13	3
Suburban	3	1
Rural	2	0
Other	1	1
**Highest Family Medicine Qualification**		
Final	11	4
Intermediate	8	1
**Country of Practice**		
Ghana	5	2
Nigeria	5	1
South Africa	2	1
Uganda	2	–
Ethiopia	1	
Liberia	1	–
Rwanda/DRC	1	–
Sierra Leone	1	1
The Gambia	1	–

The rest were at the middle level of training qualifications in Family Medicine. Of the respondents, 15 (78.9%) had completed at least 10 years of practicing the specialty with 45 years as the longest duration of practice. Four (4) respondents had been practicing for <10 years with two of them having practiced for a little over 1 year. A majority (13 representing 68.4%) of the respondents practiced in urban areas. Five (5) respondents practiced in suburban and rural settings.

The core values that were selected were Collaborative Care (Option 3), Comprehensive Care (Option 6), Continuity of Care (Option 7), Life-long learning (Option 18), and Patient centered care (Option 19). This list represents the frequency with which these core values were selected by the participants. The list of options is presented as Figure 1 below.

None of the core values were merged by the participants. A summary of five (5) comments given by respondents is presented as follows:

“*Patient-centeredness encourages compassionate and comprehensive care. All the above values confirm Family Medicine as the leading edge with large doses of humanness. Thanks*.”;

“*Family medicine must have a human face and human touch bcuz we are closest to the family yea society and the comm. and the World*”; and

“*I believe we need 2 different lists to separately rank the core values and the tasks of African FP. It is challenging to do it in one list*”.

In round 2, only five of the participants responded to the questionnaire, with a non-response rate of 63.2%. The age range of respondents in this round was 35 to 54 years with a male: female ratio of 0.7:1.0. Four of these respondents (80%) had completed terminal qualifications in Family Medicine and had been in practice for between 2 and 10 years. They were mainly urban and suburban practitioners (80%) with country of practice based in West or South Africa.

The five core values ranked in order of importance/significance were (1) Comprehensive Care, (2) Continuity of Care, (3) Collaboration (4) Patient-centered Care, and (5) Life-long Learning, This is the same order in frequency of responses from round one were recorded. This sample was however considered as too small, not adequately experienced and not truly representative of the population under review to be used to rank the selected core values from the first round.

## Discussion

This study was designed to explore the core value system of Family Physicians and General Practitioners practicing in Africa. The cohort of participants was balanced in gender with overall male:female ratio of 1.7:1.0. This varies from studies among medical practitioners which is usually male dominated (10). The views of female practitioners are thus fairly well-represented in this data.

### Comprehensive Care

Comprehensive care was considered as relevant because most African practitioners work in environments where there is scarcity of other specialists and facilities for higher care. It is therefore the duty of the Family Physician to explore the patient thoroughly in a bid to elucidate the reason for encounter and all other healthcare needs of the patient. This was the experience from a study in Sudan where procedural skills and hospital care were identified as crucial to the practice of Family Physicians because these skills are deployed regularly in their practice (4). Further to this, several diagnoses which may require referral have to be managed initially at the first facility where the patient reports to, before referral processes are put in place to effect transfer of patients. These challenges are made more pronounced in African health systems where out-of-pocket payments are rife compared to insurance-covered patients. This causes delays in referrals and transfers because in the absence of upfront out-of-payments, patients cannot be discharged from the referring facility. The next challenge will be a difficulty in payment for the transfer of the patient and the initial cash expenses required at the receiving hospital.

### Continuity of Care

Continuity of care is a value that patients cherish especially in patients with chronic medical conditions, elderly and adolescents. It engenders trust in the doctor-patient relationship and further supports another core value of patient-centeredness. With this attribute of repeated contact, complaints that appear to be independent of each other can be viewed in relation to each other and be defined (7). In the African context, patients do not generally register with specific primary care providers. Few countries like Ghana have tried the capitation method of health insurance to compel patients to visit specific practitioners for their healthcare needs. Largely, this has not been successful (11). Focused antenatal care has however been able to incorporate continuity of care, albeit over a short period of 6–9 months in the pregnant woman, to improve health outcomes in delivery (12). Family Physicians are encouraged in the African context to support healthcare delivery by encouraging patients to adopt the health-seeking behavior of using a designated practitioner for most of their healthcare needs. This will allow knowledge and experience from prior contacts between the practitioner and the patient to be applied during subsequent episodes of illness or health problems.

### Collaboration

Collaboration was the third most important core value listed by the experts in this study. The World Organization of Family Doctors and the World Health Organization have descried the five-star doctor concept (13). Effective communication is one of the five stars prescribed under this model as a skill recommended for Family Physicians. This skill is cardinal to the ability to collaborate with key stakeholders.

A collaborative practice-ready health worker is someone who has learned how to work in an interprofessional team and is competent in doing so. Collaborative practice happens when multiple health workers from different professional backgrounds work together with patients, families, carers, and communities to deliver the highest quality of care. It allows health workers to engage any individual whose skills can help achieve local health goals (14).

Collaboration is expected between multiple stakeholders including professionals (clinicians and non-clinical professions), managers, patients, producers of healthcare products, scientists, and governments/policy makers (15). This has an impact on the quality of care that a patient receives each time they engage with the healthcare delivery system. Collaborative care is crucial in the African context because resources are usually not concentrated at all facilities. Healthcare institutions tend to “specialise” based on the expertise and equipment available. Collaboration therefore ensures that patients receive the best of service available in their environments. Family Physicians are expected to facilitate collaboration between their patients and other stakeholders to promote their health and prevent disease. Collaborative practice strengthens health systems and improves health outcomes.

### Patient Centeredness

**Figure 3:**
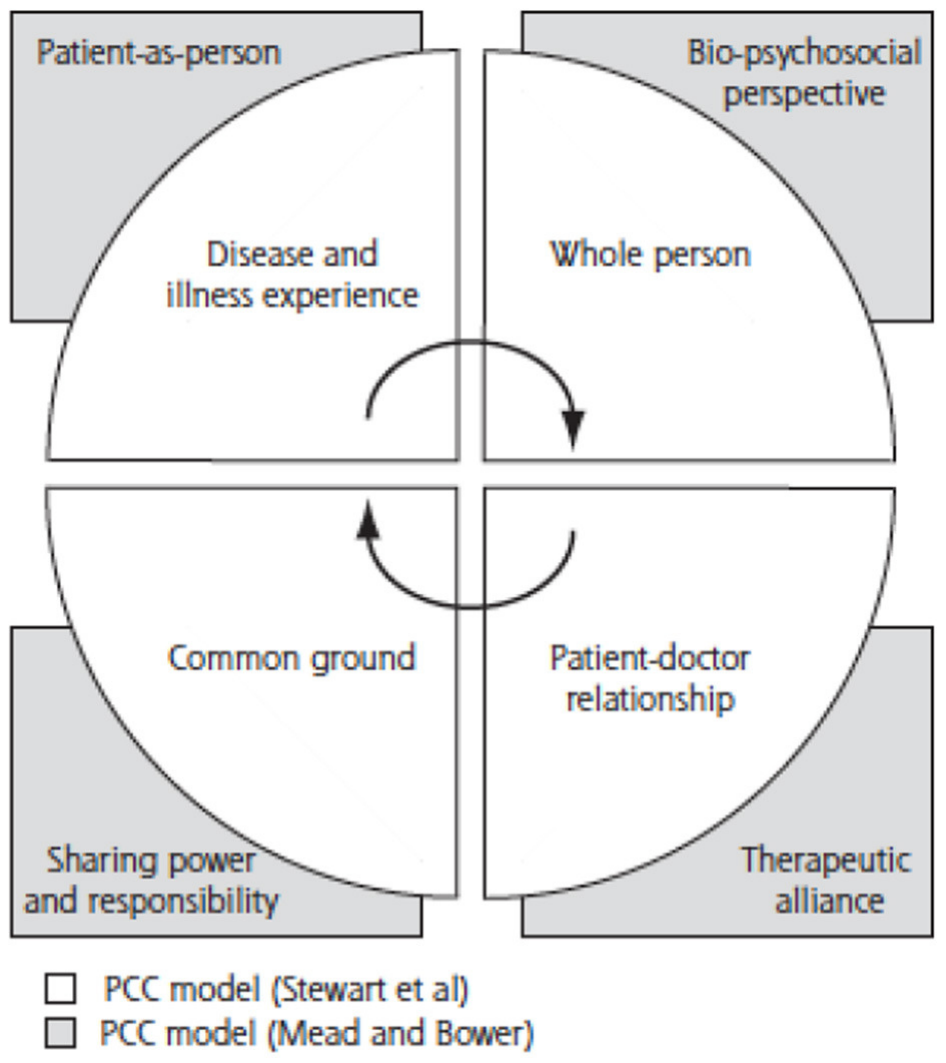
Conceptual Framework of Patient-Centered Care (18).

Patient-centered care (PCC) can be described simply as helping patients to be more active in consultations. This changes centuries of physician-dominated dialogues to those that engage patients as active participants (16). In the setting of primary care, and specifically family practice, patient-centered concepts incorporate six interactive components. The first component is the physician's exploration of both the patients' disease and dimensions of the illness experience including: their feelings about being ill, their ideas about what is wrong with them, the impact of the problem on their daily functioning, and their expectations of what should be done. The second component is the physician's understanding of the whole person. The third component is the patient and physician finding common ground regarding management. In the fourth component the physician incorporates prevention and health promotion into the visit. The fifth component is the enhancement of the patient-physician relationship. Finally, the sixth component requires that PCC should be realistic (10). In the recent past, this model has been merged with that of Mead and Bower (17) to create a new conceptual framework from the four (4) dimensions common to Stewart et al. and Mead and Bower's review: (1) disease and illness experience (patient-as-person in Mead and Bower's model), (2) whole person (biopsychosocial perspective), (3) common ground (sharing power and responsibility), and (4) patient-doctor relationship (therapeutic alliance) (18). This is illustrated in Figure 3 below.

PCC was selected as a core value in the African context. This is interesting because the biopsychosocial model of healthcare is amply demonstrated in the African context where personal beliefs, culture and tradition affect health-seeking behavior and health practices significantly. It is thus imperative for the African Family Physician to manage each patient using principles of patient centeredness which is enshrined in their training. Globally, patient centeredness is a shared core value among Family Physicians (1, 4, 6, 7).

### Life-Long Learning

The fifth core value and task selected for Family Physicians in the African context was Life-long learning. Life-long learning is a “continuously supportive process which invigorates and empowers people to obtain all the knowledge, attitudes, and skills” individuals will require all through their lifetime and apply them with confidence, creativity, and happiness (19). There is a general consensus among medical practitioners that the knowledge and skills acquired at the end of formal undergraduate and postgraduate professional medical education are not sufficient to sustain competence and performance over a career. Therefore, physicians are expected to effectively engage in life-long learning strategies (20). This core value has become more imperative because there is increasing scrutiny of professional conduct and public concerns related to the quality of care, safety of the health system, and the frequency of adverse events/medical errors. In several countries in Africa, continuing professional development is mandatory and all practicing physicians must demonstrate evidence of educational activity before annual renewal of their licenses. This is supportive of the core value of Family Physicians in Africa because of the wide breath of practice and the need to stay abreast. Family Physicians also require knowledge about sources and availability of new treatment modalities to be able to advice their patients appropriately. Family Physicians are encouraged to pursue life-long learning through identification their own learning needs, meeting those needs at their own pace and using their workplace as a key source of learning. There have been reports of burnout among doctors who do not practice life-long learning (21).

The Core values and tasks of the Family Physician have far reaching effects. For example, they can become the standard by which performance is measured by medical regulators. They can also be used to assess quality of care rendered by health insurance companies during reimbursement and renewal of contracts with practitioners (16). Training physicians to be more mindful, informative, and empathetic transforms their role from one characterized by authority to one that has the goals of partnership, solidarity, empathy, and collaboration.

Medical training institutions at the undergraduate and postgraduate levels should incorporate these core values into their curricula. Trainers should develop the capacity to describe these core values and find innovative ways of applying them in teaching to translate into the practice of their Residents.

Life-long learning skills and strategies should be actively explored at all times. Flexibility of learning methods should also be encouraged as alternatives to didactic learning which most continuing medical education programmes are used to delivering. In these times, technology should also be incorporated in the application and practice of Family Physicians to assure these core values in their clients. Finally, the authors recognize that core values do not always need to be ranked. They are a collective set of behaviors and actions that will define the African Family Physician. We are in no way suggesting that one core value is more important than the other because one was listed before the other. We expect that this is the beginning of refining the practice of Family Physicians and General Practitioners to improve patient outcomes. We suggest that these values should be assessed at all levels of training and practice of prospective Family Physicians to ensure that it is not lost after training.

This study was limited by the high non-response rate of the second round of the study. This may have altered the ranking of the five selected core values in the second round. The nascent presence of Family Medicine in Central Africa is reflected in this study with no participation from that region. Finally, lusaphone and francophone African countries are also burgeoning when it comes to practice and training of Family Physicians. Their opinions are also not captured in this survey. There was also a higher number of participants from the West African sub-region—Ghana and Nigeria. Future studies on this subject with larger sample size is recommended.

## Conclusion

This study reports that the most important core values to African Family Physicians are Comprehensiveness of Care, Continuity of Care, Collaborative Care, Patient centered care and Life-long learning. These core values should guide the development of curricula, standardization of training methods and creation of benchmarks for standards of practice for the specialty in Africa.

## Data Availability Statement

The raw data supporting the conclusions of this article will be made available by the authors, without undue reservation.

## Author Contributions

HL was involved with the development of concept, data collection, and writing the manuscript. DN was involved with collection of data and writing the manuscript. All authors contributed to the article and approved the submitted version.

## Conflict of Interest

The authors declare that the research was conducted in the absence of any commercial or financial relationships that could be construed as a potential conflict of interest.
